# Apolipoprotein E (APOE) genotyping in the Northern Ireland COhort for the Longitudinal study of Ageing (NICOLA)

**DOI:** 10.1186/s13104-026-07887-3

**Published:** 2026-05-23

**Authors:** Claire Hill, Claire Potter, Laura Smyth, Jill Kilner, Katie Quinn, Angela Scott, Charlotte Neville, Frank Kee, Bernadette McGuinness, Amy Jayne McKnight

**Affiliations:** Centre for Public Health, Queen’s University Belfast, Belfast, UK

**Keywords:** APOE, Genotyping, Ageing, NICOLA

## Abstract

**Objectives:**

Apolipoprotein E (APOE) status was determined by directly genotyping the genetic variants, rs7412 and rs429358 in the Northern Ireland COhort for the Longitudinal study of Ageing (NICOLA), harnessing blood-derived DNA. This data was generated on a subset of participants (*n* = 2947) within the NICOLA cohort.

**Data description:**

The NICOLA study recruited approximately 8500 people from across Northern Ireland, with individuals recruited representative of the Northern Ireland population. At baseline, 45% of participants were male and 55% were female. Adults were recruited over the age of 50, with approximately 50% of participants aged between 50 and 65, and 10% of participants over the age of 80. APOE status was determined for 2947 individuals (47.6% male). The APOE protein has three major isoforms (ɛ2, ɛ3, and ɛ4), determined from the direct genotyping of two single nucleotide polymorphisms (SNPs) SNPs, rs7412 and rs429358.

## Objective

Harnessing participants from the Northern Ireland COhort for the Longitudinal study of Ageing (NICOLA) [1–3], blood derived DNA collected from individuals at Wave 1 was genotyped for rs7412 and rs429358, enabling Apolipoprotein E (APOE) status to be determined. The data presented here is associated with the larger NICOLA study, led by the Centre for Public Health at Queen’s University Belfast (https://www.qub.ac.uk/sites/NICOLA/). Participants underwent detailed assessments, including computer-assisted interviews, self-completed questionnaires, physical measurements, and biological sample collection [4, 5].

The NICOLA study seeks to advance our understanding of ageing and how it affects health and well-being. For NICOLA, 8,500 adults over the age of 50 were recruited from across Northern Ireland. Other household members (spouse/partner) were also able to participate, even if they were under the age of 50. 2.3% of participants were under the age of 50 at the time of recruitment. At recruitment, approximately half of the participants were aged 50–65, and 10% were over 80 years of age. The cohort was 45% male and 55% female. NICOLA is tracking participants over time to uncover factors that contribute to healthy ageing.

The health assessment data already collected and generated (such as health measures, disease diagnoses, genetic variation, epigenetic variation and epigenetic clocks) is available for use by researchers globally. The NICOLA study website contains details of all the data that is available through a fully searchable data dictionary (https://www.qub.ac.uk/sites/NICOLA/ForResearchers/).

## Data description

The NICOLA study aims to identify risk factors for age-related diseases and build evidence to inform policies and interventions to encourage healthy ageing in the Northern Ireland population. Findings from this study can also be compared with similar cohorts worldwide, to advance the healthy aging of our global population. Wave 1 was completed for baseline data [5], with Wave 2 completed, and Wave 3 ongoing at time of this publication, with a repeat health assessment and biological sample collection.

All supplementary figures and supplementary tables are available at the following DOI: 10.17605/OSF.IO/ZD37J (NICOLA Data Products - associated files, APOE data [6], Table 1, Data File 1).

To improve accuracy and interpretation, instead of using imputation, NICOLA Wave 1 participants who had attended the health assessment and consented to venepuncture and genetic analysis were directly genotyped for APOE rs429358 and rs7412. APOE status was determined for 2,947 individuals. DNA used for this assessment were derived from blood samples collected during the Wave 1 health assessment for 3,514 participants [5]. Blood sample handling and DNA extraction were carried out as described previously [5]. The APOE data sample subset is 47.6% male.

Genotype calls were generated by the Queen’s University Belfast Genomics Core Technology Unit using the Taqman kit for APOE from Thermo Fisher. The two assays were rs7412 (kit no C_904973_10) and rs429358 (kit no C_3084793_20). The plate was read on the ABI 7900 HT system. A non-template control was included in each assay.

APOE status was determined via the following algorithm (visually outlined in Supplementary Figs. 1–2):

- ɛ2/ ɛ2: rs429358 T/T, rs7412 T/T,
- ɛ2/ ɛ3: rs429358 T/T, rs7412 C/T,
- ɛ2/ ɛ4: rs429358 C/T, rs7412 C/T,
- ɛ3/ ɛ3: rs429358 T/T, rs7412 C/C,
- ɛ3/ ɛ4: rs429358 C/T, rs7412 C/C,
- ɛ4/ ɛ4: rs429358 C/C, rs7412 C/C.

Analyses was ran across nine plates. Blind duplicates were used (*N* = 32 samples), four duplicates per plates (for plates 1–8), with all duplicates matching. Water replicates were used (four across plates 2–8, three in plate 1 and one in plate 9), all yielding undetermined APOE status. Blanks were assessed (four in plates 5–8), all yielding undetermined APOE status. Non-template controls were used (three in plate 1, 4 in plates 2–4, six in plate 4, 2 in plates 5–8 and one in plate 9), all yielding undetermined APOE status.

Age and gender breakdowns based on APOE status and APOE ɛ4 status are shown in Supplementary Tables 1–3. In the dataframes, e represents ɛ. Our data reflects patterns in recent pooled analysis of community dwellers [7]. Please note that gender in this instance refers to self-reported gender in the self-completed computer assisted personal interview (CAPI).

**Table 1:** Overview of data files/data sets

Label	Name of data file/data set	File types (file extension)	Data repository and identifier (DOI or accession number)
Data set 1	APOE data / NICOLA_APOE_filtered_151124_AP_ID_2947.csv	.csv	Open Science Framework *-* DOI: 10.17605/OSF.IO/ZD37J [6]
Data file 1	APOE data /APOE_NICOLA_datanote__supplementary_090126.pdf	.pdf	Open Science Framework - DOI: 10.17605/OSF.IO/ZD37J [6]

## Limitations

Clearly state the limitations of the data. This could be any shortcoming that occurred during data collection, small sample sizes (which prevented the data to be used as part of a research paper) or outdated data among others. We believe all sound data is valuable and deserves publication. Honest and transparent description of any limitations will not affect editorial assessment. Limitations may be presented in bullet point format.

- APOE status was only determined for a subset of individuals for which good quality DNA was available for genotyping, approximately 75% of the NICOLA cohort does not have this information available. There is scope to increase this in further waves of the NICOLA study.

## Data Availability

The data described in this Data note can be freely and openly accessed on the Open Science Framework under DOI 10.17605/OSF.IO/ZD37J (NICOLA Data Products - associated files, APOE data) [6]. Please see Table 1 for details.

## Abbreviations

NICOLA: Northern Ireland COhort for the Longitudinal study of Ageing
APOE: Apolipoprotein E
DNA: Deoxyribose nucleic acid
SNPs: Single nucleotide polymorphism
CAPI: Computer assisted personal interview
